# Cancer-Associated Fibroblasts and Prostaglandin E_2_ Signaling in Gastric Cancer: Immunometabolic Mechanisms and Implications for Immunotherapy Resistance

**DOI:** 10.3390/ijms27219544

**Published:** 2026-10-25

**Authors:** Chongxuan Xu, Yihan Wang, Qiaoqiao Zhang, Yihe Dong, Mengjing Yuan, Dongxuan Li, Shiyang Deng, Jiang Du

**Affiliations:** 1 Department of Pathology, The First Affiliated Hospital of China Medical University, Shenyang 110001, China; 13998662537@163.com (C.X.);; 2 The Fourth Clinical College, China Medical University, Shenyang 110122, China; 3 School of Stomatology, China Medical University, Shenyang 110002, China

**Keywords:** gastric cancer, cancer-associated fibroblasts, prostaglandin E_2_, immunotherapy resistance, tumor microenvironment, CD8^+^ T cells

## Abstract

Cancer-associated fibroblasts (CAFs) and prostaglandin E_2_ (PGE_2_) signaling have each been implicated in resistance to immune checkpoint inhibitors (ICIs) in gastric cancer. CAFs are associated with immune exclusion, whereas PGE_2_ can suppress CD8^+^ T-cell responses through prostaglandin E receptors 2 and 4 (EP2 and EP4) and alter dendritic-cell and myeloid-cell function. Whether PGE_2_ produced by CAFs directly contributes to ICI resistance in gastric cancer, however, remains unclear. To distinguish association from functional involvement, we synthesize the available literature into a conceptual continuum describing the possible contribution of PGE_2_ to CAF-associated immunotherapy resistance. At the Model A end, PGE_2_ may accompany CAF activation or local inflammation, whereas other CAF-associated mechanisms, including extracellular matrix remodeling, lactate, and CXCL12 signaling, may account for much of the resistance phenotype. At the Model B end, PGE_2_ may make an identifiable functional contribution to resistance beyond these alternative mechanisms, although the cellular source of the relevant PGE_2_ remains unresolved. These models represent interpretive endpoints derived from the available evidence rather than experimentally validated resistance subtypes or an established CAF-derived PGE_2_ pathway. Clinical findings with the EP4 antagonist ONO-4578 support the therapeutic potential of receptor blockade but do not identify the cellular source of PGE_2_ involved. Key unresolved questions concern the cellular sources of PGE_2_, its relationship with immune exclusion, and whether PGE_2_-related markers improve the prediction of ICI response beyond established indicators.

## 1. Introduction

Gastric cancer remains a major cause of cancer incidence and mortality worldwide. Immune checkpoint inhibitors (ICIs), particularly antibodies targeting PD-1 or PD-L1, have improved outcomes for some patients with advanced disease. However, response rates to ICI monotherapy remain limited in unselected populations and later-line settings. Although adding ICIs to first-line chemotherapy has improved response rates, both primary and acquired resistance remain common [1]. Intrinsic tumor features, including microsatellite instability and Epstein–Barr virus (EBV) status, explain only part of the variation in treatment response. Interactions between stromal and immune cells in the tumor microenvironment (TME), particularly stromal remodeling accompanied by immune exclusion, may also influence ICI efficacy, although their contribution has not been fully established [1,2,3]. How stromal inflammation contributes to immune exclusion and ICI resistance in gastric cancer therefore remains incompletely understood.

**Figure 1:**
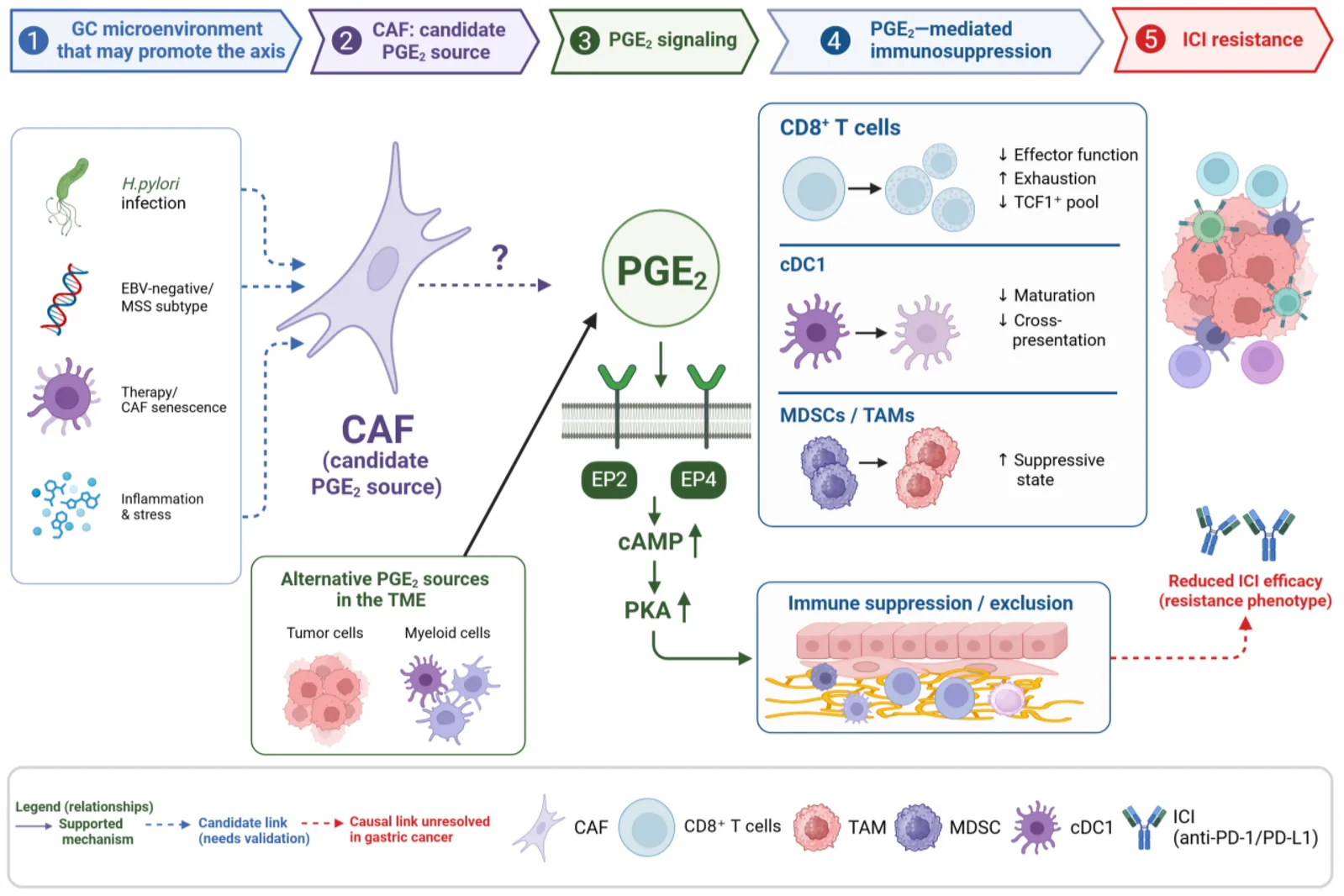
Candidate model of the CAF–PGE_2_ axis in gastric cancer immunotherapy resistance. Gastric cancer CAFs may contribute to ICI resistance through PGE_2_-dependent and PGE_2_-independent mechanisms, including extracellular matrix remodeling, lactate production, and other soluble mediators. PGE_2_ signaling through EP2/EP4 may impair CD8^+^ T-cell function and remodel dendritic-cell and myeloid-cell networks. Solid arrows denote relationships supported at the indicated pathway step; green dashed arrows denote the candidate CAF source link; the red dashed arrow indicates that the complete source-specific causal chain leading to reduced ICI efficacy has not been established in gastric cancer. Tumor cells and myeloid cells are alternative sources of PGE_2_. CAF, cancer-associated fibroblast; DC, dendritic cell; ICI, immune checkpoint inhibitor; PGE_2_, prostaglandin E_2_. The purple dashed arrow with a question mark indicates the proposed CAF-to-PGE_2_ source relationship. Although gastric cancer-derived fibroblasts can secrete PGE_2_ in vitro, their contribution to intratumoral PGE_2_ and ICI resistance in vivo remains to be established.

Cancer-associated fibroblasts (CAFs) are prominent stromal constituents of solid tumors. In gastric cancer, CAF populations vary in composition, functional state, and immunological activity. CAF composition has been associated with ICI response, and CAFs may promote immune exclusion through extracellular matrix (ECM) remodeling, CXCL12, lactate, and other soluble mediators [3,4,5]. Prostaglandin E_2_ (PGE_2_), a bioactive lipid mediator, can suppress T-cell activity through EP2 and EP4 and also affects dendritic cells (DCs) and myeloid-cell populations [6]. In EBV-negative gastric cancer, reduced CD8^+^ T-cell infiltration has been associated with increased COX-2/PGE_2_ pathway activity [7]. These findings suggest that CAF-associated stromal remodeling and PGE_2_-mediated immunosuppression may both be relevant to ICI response. However, the cellular origin of PGE_2_ and its functional relationship with CAF activity in gastric cancer remain unclear (Figure 1).

**Figure 2:**
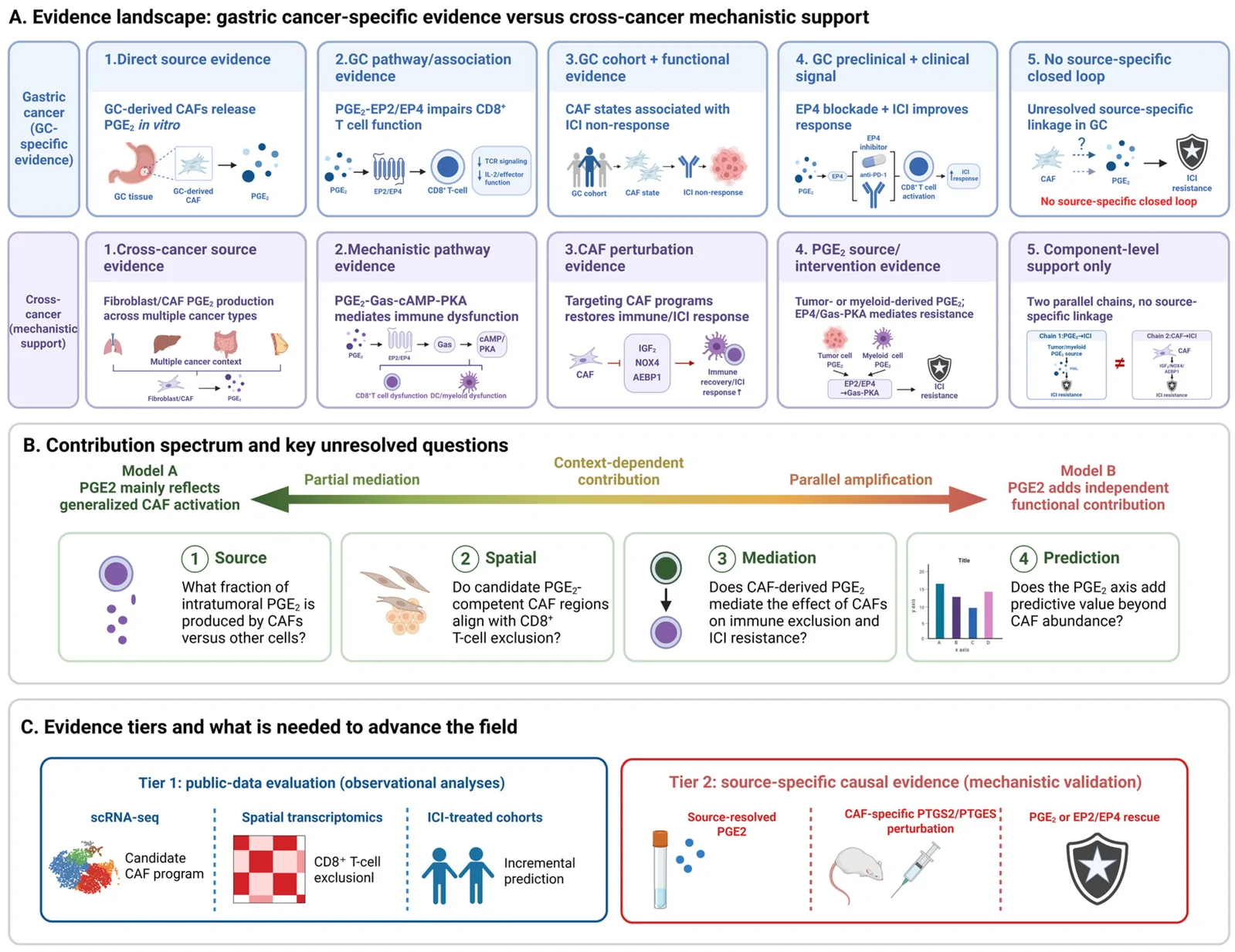
Evidence dimensions, a contribution spectrum, and testable predictions for the candidate CAF–PGE_2_ axis. (**A**) Direct evidence in gastric cancer and cross-cancer mechanistic support are presented separately. Cross-cancer evidence for the complete axis is limited to individual components; no source-specific closed loop has been validated. (**B**) Model A and Model B are conceptual endpoints of a continuum: PGE_2_ may largely accompany generalized CAF activation, contribute independently, or show partial mediation, context-dependent contribution, or parallel amplification between these endpoints. (**C**) Public data can assess a candidate PGE_2_-competent CAF program, its spatial contribution, and ICI outcome prediction (Tier 1); source-resolved metabolite measurement, perturbation, and rescue are required for causal attribution (Tier 2).

This review examines potential sources of PGE_2_ in gastric cancer, its spatial relationship with immune exclusion, and its possible contribution to ICI resistance. We also assess whether combined CAF- and PGE_2_-related features might provide predictive information beyond CAF abundance, PD-L1, MSI/EBV status, and conventional clinical variables (Figure 2). Here, a ‘candidate CAF program with PGE_2_ biosynthetic potential’ refers to inflammatory CAF features accompanied by increased PTGS2/PTGES expression and reduced HPGD expression. HPGD encodes 15-hydroxyprostaglandin dehydrogenase (15-PGDH), an enzyme involved in PGE_2_ degradation. This operational definition describes a putative metabolic state, not a validated CAF subtype.

## 2. PGE_2_ Metabolic Features of CAFs in Gastric Cancer

PGE_2_ is a bioactive lipid mediator synthesized from arachidonic acid. Arachidonic acid released from membrane phospholipids is converted by cyclooxygenases (COX-1/PTGS1 and COX-2/PTGS2) into PGH_2_, which is then converted into PGE_2_ by prostaglandin E synthases, including microsomal prostaglandin E synthase-1 (mPGES-1/PTGES). Local PGE_2_ availability reflects both its synthesis and degradation. The principal PGE_2_-inactivating enzyme, 15-hydroxyprostaglandin dehydrogenase (15-PGDH, encoded by HPGD), is NAD^+^-dependent and oxidizes the 15(S)-hydroxyl group of PGE_2_ to form inactive 15-keto-PGE_2_, thereby reducing local levels of bioactive PGE_2_ [6,8,9]. In gastric cancer, reduced 15-PGDH/HPGD expression has been associated with PGE_2_ accumulation, increased FOXP3^+^ regulatory T-cell abundance, a more immunosuppressive microenvironment, and poorer prognosis. Concurrent PTGS2/PTGES upregulation and HPGD downregulation may therefore favor PGE_2_ accumulation in the TME. Together with inflammatory CAF markers, these features form the basis of our operational definition of a candidate CAF program with PGE_2_ biosynthetic potential. This designation indicates potential biosynthetic capacity, but neither identifies a validated CAF subtype nor demonstrates PGE_2_ secretion by CAFs. Tumor cells and myeloid cells can also use this pathway; transcript abundance alone cannot establish which cell population contributes most to intratumoral PGE_2_.

### 2.1. PGE_2_ Biosynthetic Potential and Immunomodulatory Functions of CAFs

Fibroblasts isolated from gastric cancer tissue can produce PGE_2_ in culture [10]. Inflammatory CAFs may express PTGS2, IL-6, IL-8, CXCL12, and related programs [11,12,13]. Epigenetic silencing of miR-149 may also contribute to the interplay between PGE_2_ and IL-6 [14]. PDPN^+^ and POSTN^+^ CAF populations have been associated with impaired CD8^+^ T-cell function or poorer immunotherapy responses [15,16]. These observations, however, do not establish whether PGE_2_ mediates the associated immune effects.

Because CAF states differ, greater CAF abundance does not necessarily increase local PGE_2_ exposure. Myofibroblastic CAFs may instead restrict immune-cell infiltration by depositing ECM [17]. Other CAF states can influence PD-L1 expression or T-cell function through IL-8 and lactate [18,19]. Interactions of macrophages or neutrophils with perivascular or stromal CAF populations provide additional routes to an immunosuppressive microenvironment [20,21,22]. CAF abundance, PGE_2_ biosynthetic potential, and immunosuppression should therefore not be treated as equivalent measures; PGE_2_-associated programs need to be interpreted alongside other CAF-mediated mechanisms.

### 2.2. PGE_2_ Secretion by Gastric Cancer–Derived Fibroblasts

Fibroblasts derived from gastric cancer tissue release PGE_2_ in culture; selective COX-2 inhibition reduces both PGE_2_ secretion and the associated invasion-promoting activity [10]. Fibroblast-derived PGE_2_ has also been reported in lung fibroblast studies and other tumor models [9,23]. Tumor cells and myeloid cells can likewise produce immunologically active PGE_2_ [24,25,26]. Thus, the direct evidence in gastric cancer primarily concerns PGE_2_ secretion and invasion-related effects in vitro. It does not yet quantify the contribution of CAFs to intratumoral PGE_2_ in vivo or establish a CAF-derived PGE_2_ mechanism of ICI resistance.

## 3. CAF–PGE_2_-Related Changes Across Gastric Cancer Contexts

Infection, molecular subtype, treatment-related stress, and persistent inflammatory signaling may favor inflammatory stromal states or PGE_2_-associated metabolic changes in gastric cancer. These contexts help explain differences among studies (Table 1 and Table 2), although the identity of the PGE_2_-producing cell populations remains unknown.

### 3.1. Chronic Helicobacter pylori Infection

*Helicobacter pylori*-associated signaling can activate NF-κB/AP-1 and COX-2 [43,44]. Stromal COLEC12 expression and related CAF states suggest that infection may alter PGE_2_-associated signaling and stromal spatial organization [12,45,46]. *H. pylori* can induce a COX-2/PGE_2_–IL-8 program in gastric cancer cells [47], whereas some stromal mechanisms of immunosuppression appear to operate independently of PGE_2_ [48]. Infection-associated inflammation therefore involves both tumor and stromal compartments, and its relationship with PGE_2_ signaling needs to be considered in this broader cellular context.

### 3.2. Molecular Contexts: EBV-Negative and Microsatellite-Stable Gastric Cancer

EBV-positive gastric cancers have been associated with a more inflamed immune microenvironment, including greater CD8^+^ T-cell infiltration [7]. By contrast, EBV-negative tumors have been reported to show fewer infiltrating CD8^+^ T cells and higher COX-2/PGE_2_ pathway expression; one study reported approximately 32-fold higher PTGS2 transcript levels [7]. Microsatellite-stable (MSS) gastric cancers frequently have a CAF-rich stroma and have been associated with primary ICI resistance [1,49]. EP4 expression in human gastric mucosal T cells also supports the capacity of gastric tissue to respond to PGE_2_ [30]. The Lauren classification categorizes gastric adenocarcinoma into intestinal, diffuse, and mixed histological types. The intestinal type typically forms glandular or papillary structures and is often associated with *H. pylori* infection and intestinal metaplasia; the diffuse type comprises poorly cohesive or signet-ring cells, shows infiltrative growth, and has a propensity for peritoneal dissemination; the mixed type has features of both. The prominent stromal component of diffuse-type tumors suggests a possible relationship between histological subtype and CAF composition. Lauren classification is distinct from EBV and MSI/MSS molecular classification. Together, these findings provide relevant contexts for considering PGE_2_ production and spatial organization, but subtype associations alone do not demonstrate that CAFs produce PGE_2_.

### 3.3. Treatment-Induced CAF Senescence and the Senescence-Associated Secretory Phenotype

Treatment-related stress can induce fibroblast senescence and a senescence-associated secretory phenotype (SASP) containing PGE_2_, IL-6, and matrix metalloproteinases. Experimental findings implicate COX-2/PGE_2_ signaling and MMP–NF-κB/COX-2 mechanisms in these changes [50,51,52]. Thus, treatment-induced stromal senescence may link PGE_2_ to microenvironmental remodeling alongside other SASP mediators.

### 3.4. Inflammatory Feedback and Maintenance Signals

IL-1α/β, TGF-β-associated programs, and reduced 15-PGDH activity may influence CAF activation or tissue PGE_2_ accumulation [53,54,55]. Because these factors affect stromal activation and PGE_2_ metabolism through different processes, they may jointly sustain an inflammatory microenvironment.

Taken together, findings on infection, molecular subtype, treatment-related stress, and persistent inflammation place PGE_2_-associated metabolic changes and stromal remodeling in distinct gastric cancer contexts. These relationships are context-dependent, and no single clinical or molecular feature reliably identifies a specific CAF metabolic program.

## 4. PGE_2_ as an Immunometabolic Signal

### 4.1. EP2/EP4–cAMP–PKA Signaling

In the immunosuppressive contexts discussed here, PGE_2_ acts predominantly through EP2 and EP4, activating the Gαs–cAMP–protein kinase A (PKA) pathway. This signaling can suppress proximal T-cell receptor (TCR) signal transduction and NFAT/AP-1-dependent effector programs. It can also impair cytotoxic T-lymphocyte adhesion and motility [6,55,56]. In experimental systems, selective activation of Gαs–PKA signaling in CD8^+^ T cells is sufficient to induce T-cell dysfunction and immunotherapy failure [33]. Human gastric mucosal T cells also express EP4 [30]. Together, these observations support the mechanistic relevance of PGE_2_-mediated T-cell suppression while providing tissue-specific evidence of receptor expression.

### 4.2. CD8^+^ T-Cell Dysfunction

PGE_2_ can impair CD8^+^ T-cell responses during activation, expansion, and maintenance of effector function. Early in activation, it suppresses proximal TCR signaling and immunological synapse formation, reducing stable contact with target cells and cytotoxic killing [55,56,57]. During expansion, PGE_2_ disrupts IL-2-related signaling and limits the effector expansion of TCF1^+^ stem-like CD8^+^ T cells [31,57,58]. The clonal expansion observed after PD-1 blockade depends on precursors within this population; a reduced precursor pool may therefore limit reinvigoration. Prolonged PGE_2_ exposure has been associated with higher expression of PD-1, TIM-3, and TIGIT [33,59], as well as impaired mitochondrial function and metabolic adaptation [57,60,61]. Some PGE_2_ effects occur independently of PD-1/PD-L1 signaling [31]. Together, these mechanisms suggest that PGE_2_ can compromise both the initiation and persistence of T-cell responses, providing a rationale for combination strategies beyond PD-1/PD-L1 blockade alone.

### 4.3. Dendritic, Myeloid, and Regulatory Immune Cells

PGE_2_ can disrupt programs associated with IRF8, CXCL9, and IL-12 in type 1 conventional dendritic cells (cDC1s) [32,62,63], reduce NK–cDC1 recruitment [64], and promote the development of myeloid-derived suppressor cells [65,66,67]. Conversely, EP2/EP4 blockade can reverse suppressive myeloid states [40,68,69]. Together, these effects may compromise antigen presentation, T-cell recruitment, and sustained effector activity. Responses of regulatory T cells and natural killer cells to PGE_2_ depend on the tissue, dose, and receptor involved [70,71,72,73,74,75]. The net immunological effect of PGE_2_ therefore depends on the cellular and tissue context.

## 5. CAF–PGE_2_ Signaling and ICI Resistance in Gastric Cancer: Published Findings and Conceptual Models

### 5.1. Immunosuppressive Mechanisms Identified Across Tumor Types and in Gastric Cancer

Studies in several tumor types have linked PGE_2_ signaling and CAF-associated programs to impaired antitumor immunity, although the relevant mechanisms differ. In melanoma and lung cancer models, tumor-cell prostaglandin synthesis has been associated with immunosuppression and resistance to immunotherapy [25,26]. Colorectal cancer studies have shown that EP4 signaling maintains suppressive myeloid states and that EP4 antagonism can improve responses to anti-PD-1 therapy [69]. Similarly, Gαs–PKA activation in CD8^+^ T cells can promote T-cell dysfunction and immunotherapy failure [33]. Independent studies have implicated CAF-associated IGF2, NOX4, and AEBP1 programs in altered immune-cell recruitment and function [35,36,37]. More directly linking CAFs to PGE_2_, neuroblastoma models show that mPGES-1 inhibition reduces CAF-derived PGE_2_, accompanied by reduced tumor growth and changes in macrophage states [9]. In breast cancer, however, blocking PGE_2_ receptor signaling in CAFs has different effects on primary tumor growth and metastasis [76]. Taken together, these findings indicate that the source and recipient of PGE_2_, as well as tumor context, are central to interpreting CAF–PGE_2_ interactions. Findings from other tumor types cannot be assumed to establish the same mechanism in gastric cancer.

In gastric cancer, clinical and functional findings implicate several CAF states in ICI resistance. Cohort studies have associated ACTA2 expression and fibroblast abundance scores with poorer outcomes or ICI nonresponse [2,3]. Associations with immune suppression or ICI response have also been reported for DCN^+^, PDE5A^+^, SERPINE2-related, CCL2^+^, and FAP^+^ CAF populations or stromal states [5,34,49,77,78], while CCDC80^+^, CTHRC1^+^, and angiogenesis-associated CAF programs have been linked to immune dysfunction [79,80,81]. PDPN^+^ CAFs have been associated with lower pathological complete response rates after neoadjuvant anti-PD-1 therapy, and related models have shown reduced immune activation [15]. Beyond these associations, functional studies implicate CAF-derived lactate in anti-PD-1 resistance through lactylation-associated signaling [19] and link POSTN^+^ CAFs to CD8^+^ T-cell exhaustion and immunosuppression during peritoneal metastasis [16].

The studies summarized above support two related but distinct observations: PGE_2_ can suppress antitumor immunity, and several CAF-associated mechanisms are implicated in gastric cancer ICI resistance. Neither finding alone establishes that CAF-derived PGE_2_ causes resistance in gastric cancer. CAFs can act through PGE_2_-independent pathways, while tumor and myeloid cells may also produce PGE_2_. This distinction provides the rationale for considering whether PGE_2_ simply accompanies CAF activation or contributes functionally to resistance.

### 5.2. The Role of PGE_2_ in Gastric Cancer ICI Resistance: Models A and B

CAFs and PGE_2_ may both influence the immunosuppressive microenvironment of gastric cancer. Their association, however, does not establish that PGE_2_ released by CAFs directly causes ICI resistance. We therefore introduce Models A and B as conceptual endpoints describing the possible functional contribution of PGE_2_ within CAF-associated resistance.

At the Model A end, PGE_2_ primarily accompanies CAF activation or local inflammation. ICI resistance may instead be explained largely by other CAF-associated mechanisms, including extracellular matrix (ECM) remodeling, lactate, and CXCL12. Accordingly, increased PGE_2_ pathway activity does not in itself establish PGE_2_ as a major driver of resistance. In this conceptual setting, its independent contribution may be limited or difficult to distinguish from that of the broader CAF-associated program.

At the Model B end, PGE_2_ still makes an identifiable functional contribution to ICI resistance after other CAF-associated mechanisms, such as ECM remodeling and lactate, are taken into account. PGE_2_ may therefore contribute to immune-cell dysfunction and resistance rather than merely accompany them. CAFs may provide some of the PGE_2_ involved, but evidence of a PGE_2_-dependent effect does not identify its cellular source. Source attribution and functional mediation remain separate questions.

Models A and B do not represent fixed CAF subtypes, patient categories, or mutually exclusive resistance mechanisms. Instead, they mark the ends of a continuum in the possible functional contribution of PGE_2_ to ICI resistance. Intermediate states may involve partial mediation by PGE_2_ or amplification of other CAF-associated mechanisms. The continuum therefore concerns the contribution of PGE_2_ to resistance, not a positive-feedback circuit between the two models.

Importantly, this conceptual continuum should not be confused with the strength of the available evidence. A lack of functional studies does not establish that PGE_2_ has no relevant role and cannot, by itself, justify assigning a study to Model A. Conversely, concurrent CAF enrichment and increased PGE_2_ pathway activity do not establish Model B. These endpoints offer a way to interpret published findings without treating either model as a validated sample classification.

The models thus focus attention on a central distinction: is PGE_2_ merely associated with CAF-related immunosuppression, or does it make an additional functional contribution to ICI resistance in gastric cancer? This question is also relevant to the interpretation of PGE_2_ receptor-directed therapies and candidate biomarkers (Table 1, Figure 2, and Supplementary Table S3).

## 6. Translational Implications of Targeting the CAF–PGE_2_ Pathway

### 6.1. EP4 Blockade

In gastric cancer models, the EP4 antagonist YY001 has been reported to enhance responses to PD-1 blockade, accompanied by increased infiltration of antigen-presenting cells, T cells, and M1-like macrophages [29]. Early clinical findings have also reported activity with ONO-4578 combined with nivolumab [38]. In the randomized, double-blind phase II ONO-4578-08 trial, first-line treatment with ONO-4578 or placebo, each combined with nivolumab and chemotherapy, was evaluated in patients with HER2-negative advanced gastric or gastroesophageal junction cancer. Median progression-free survival (PFS) was 9.0 months in the ONO-4578 group and 6.9 months in the placebo group (hazard ratio, 0.67; 90% confidence interval, 0.48–0.92) [39]. In the subgroup with a programmed death-ligand 1 (PD-L1) combined positive score (CPS) ≥ 1, median PFS was 9.9 versus 5.7 months (hazard ratio, 0.52; 95% confidence interval, 0.34–0.79) [39].

These findings provide a clinical signal supporting the therapeutic potential of EP4 blockade, but they do not establish that CAF-derived PGE_2_ mediates the observed treatment effect. EP4 antagonism acts at the receptor level and does not, by itself, identify the cellular source of the PGE_2_ involved. Moreover, EP2 and EP4 share downstream Gαs–cAMP–PKA signaling, so selective EP4 blockade may not fully inhibit all PGE_2_-mediated effects [33,40,82]. Thus, the current findings support the therapeutic relevance of PGE_2_ receptor signaling while leaving the source-specific contribution of CAF-derived PGE_2_ unresolved (Table 3).

### 6.2. Inhibition of COX-2 or mPGES-1

Inhibition of cyclooxygenase-2 (COX-2) or microsomal prostaglandin E synthase-1 (mPGES-1) provides an upstream strategy for reducing PGE_2_ synthesis. In gastric cancer, COX-2 expression has been associated with prostaglandin biosynthesis and angiogenesis [87]. Experimental studies in other tumor types have reported that tumor-cell PTGES deletion or COX-2 inhibition can improve CD8^+^ T-cell and dendritic-cell states and enhance responses to anti-PD-1 treatment [25,26]. Neuroblastoma models provide additional functional evidence implicating CAF-associated mPGES-1 in PGE_2_ production and tumor progression [9].

However, these findings do not establish the efficacy of CAF-directed COX-2 or mPGES-1 inhibition in gastric cancer. COX-2 inhibition can affect the production of multiple prostanoids, whereas mPGES-1 inhibition may offer greater selectivity for PGE_2_ synthesis while relatively preserving other prostanoids. The consequences of either intervention may depend on the cellular source of PGE_2_ and the surrounding tumor context. Consistent with this context dependence, studies in breast cancer have reported different effects of disrupting PGE_2_ signaling in CAFs on primary tumor growth and metastasis [76]. Taken together, these findings highlight an important distinction between COX-2 and mPGES-1 inhibition: while both approaches can attenuate PGE_2_ synthesis, they differ in pathway selectivity and may therefore have distinct biological consequences across tumor contexts. Their potential relevance to gastric cancer immunotherapy should consequently be interpreted in light of the cellular sources of PGE_2_ and the contribution of alternative immunosuppressive mechanisms, rather than being attributed solely to CAF-associated signaling.

## 7. Discussion

Taken together, these findings highlight an important distinction between COX-2 and mPGES-1 inhibition: while both approaches can attenuate PGE_2_ synthesis, they differ in pathway selectivity and may therefore have distinct biological consequences across tumor contexts. Their potential relevance to gastric cancer immunotherapy should consequently be interpreted in light of the cellular sources of PGE_2_ and the contribution of alternative immunosuppressive mechanisms, rather than being attributed solely to CAF-associated signaling. In parallel, PGE_2_ has been linked to suppression of CD8^+^ T-cell activity and changes in DC and myeloid-cell function through EP2/EP4 signaling. Fibroblasts isolated from gastric cancer tissue can produce PGE_2_ in vitro, while studies in several cancer types have linked PGE_2_ signaling to immunosuppression and ICI resistance. EP4 antagonist studies have also provided preliminary support for targeting this pathway. However, CAF-mediated immune regulation and PGE_2_ signaling have largely been investigated as separate mechanisms, leaving their functional connection in gastric cancer ICI resistance uncertain.

A major unresolved issue is the cellular origin of PGE_2_ in the gastric cancer TME. Some inflammatory CAF populations express genes associated with PGE_2_ biosynthesis, and fibroblasts derived from gastric cancer can release PGE_2_ in vitro. Nevertheless, tumor cells and myeloid cells are also potential sources, and their relative contributions to intratumoral PGE_2_ are unknown. A second gap concerns the relationship between CAF-associated PGE_2_ signaling and immune exclusion. CAF enrichment, stromal remodeling, and reduced CD8^+^ T-cell infiltration have been linked in gastric cancer, but ECM barriers, lactate, and other mediators may produce similar effects. The extent to which PGE_2_ contributes independently to immune exclusion, and whether this contribution varies with CAF composition or tissue context, therefore remains unclear.

Whether CAF-derived PGE_2_ contributes functionally to gastric cancer ICI resistance remains a related, but distinct, question. Existing findings support a relationship between CAFs and resistance, as well as PGE_2_-mediated suppression of antitumor immunity, but direct gastric cancer evidence linking CAF-derived PGE_2_ to ICI resistance remains limited. Models A and B help distinguish these possibilities. In Model A, PGE_2_ primarily accompanies CAF activation or inflammation, and other CAF mechanisms may account for most resistance. In Model B, PGE_2_ makes an identifiable contribution even after these mechanisms are considered, with CAFs potentially providing part of the relevant PGE_2_. These conceptual endpoints are not validated resistance subtypes. Moreover, lack of functional evidence cannot be interpreted as support for Model A.

The translational relevance of this distinction is illustrated by studies of EP4 antagonism. Gastric cancer models and clinical findings with ONO-4578 support the potential of PGE_2_ receptor-directed treatment but cannot establish that its benefit depends on CAF-derived PGE_2_. The clinical value of CAF- and PGE_2_-related features as candidate biomarkers also remains uncertain. Beyond their associations with ICI response, such features would need to offer additional predictive information relative to PD-L1, MSI/EBV status, and existing clinical indicators. Differences in molecular subtype, stromal composition, and immune state may further limit their generalizability.

Together, these findings suggest that CAF-associated programs and PGE_2_ signaling may jointly shape the immune context of gastric cancer, although the specific contribution of CAF-derived PGE_2_ remains unresolved. The principal gaps concern PGE_2_ source attribution, its role in local immune exclusion, and its functional contribution alongside other CAF-associated mechanisms. Their clinical significance also depends on whether related biomarkers provide useful predictive information and whether targeting PGE_2_ signaling benefits defined patient populations. Addressing these gaps would clarify the relevance of the candidate CAF–PGE_2_ axis to ICI resistance without presuming a source-specific causal mechanism.

## 8. Conclusions

The potential contribution of the CAF–PGE_2_ axis to immune checkpoint inhibitor (ICI) resistance in gastric cancer raises an important question about the relationship between tumor stroma and immunosuppression. Although CAFs have been linked to ICI resistance and PGE_2_ can modulate antitumor immune responses, direct causal evidence that CAF-derived PGE_2_ mediates ICI resistance in gastric cancer remains insufficient. We therefore propose Models A and B to distinguish two conceptual endpoints of PGE_2_ involvement in CAF-associated resistance. In Model A, PGE_2_ mainly accompanies CAF activation or inflammation, while other CAF-associated mechanisms may explain most of the resistance phenotype. In Model B, PGE_2_ makes an identifiable functional contribution even after these competing mechanisms are considered, with CAFs potentially serving as one source of the relevant PGE_2_. These models are not fixed resistance subtypes; they represent endpoints of a continuum that may also include intermediate states. Importantly, an association between PGE_2_ and CAF activation does not establish PGE_2_-mediated resistance, just as an absence of functional evidence cannot be taken as support for Model A. Clarifying the contribution and clinical relevance of CAF-derived PGE_2_ will help determine whether this candidate axis is a useful target for overcoming ICI resistance in gastric cancer.

## Figures and Tables

**Table 1:** Evidence grading, current interpretation, and validation thresholds for components of the CAF–PGE_2_ axis in gastric cancer.

Component	Direct Evidence in Gastric Cancer	Cross-Cancer Support	Current Interpretation and Validation Threshold
CAF → PGE_2_	Gastric cancer-derived fibroblasts secrete PGE_2_ in vitro [10]	Direct CAF PGE_2_ and inflammatory fibroblast evidence across cancer types [9,27,28]	Candidate source; requires in situ source quantification and a decrease in tissue PGE_2_ after CAF-specific intervention
PGE_2_ → CD8/DC/myeloid dysfunction	Pathway, receptor, and association evidence in gastric cancer [7,29,30]	Direct T-cell, dendritic-cell, and pathway evidence [31,32,33]	Downstream mechanism is plausible; receptor abundance, exposure, and spatial contribution require in situ validation in gastric cancer
CAF → ICI resistance	Representative cohort and functional evidence [3,15,34]	CAF-directed interventions restore immunity or ICI response [35,36,37]	Strongly supported; validated mediators include non-PGE_2_ programs such as lactate and ECM
PGE_2_ → ICI resistance	EP4 gastric cancer models and clinical combination signals [29,38,39]	Source perturbation, receptor blockade, and T-cell pathway evidence [33,40,41]	PGE_2_ is a tractable node; source-specific mediation in gastric cancer remains unresolved
Complete CAF → PGE_2_ → ICI axis	No source–mediator–efficacy closed loop in a single system	Component-level support only; no closed-loop source-specific validation	Contribution remains unresolved; requires source quantification, continuous mediation estimates, and perturbation–rescue experiments

Note: Direct gastric cancer evidence and cross-cancer mechanistic support are shown separately; the latter does not replace source-specific causal evidence in gastric cancer. For the complete-axis row, the cross-cancer column indicates component-level support only and does not assign a closed-loop rating. Study-level details are provided in Supplementary Table S3.

**Table 2:** Gastric cancer-specific factors potentially associated with the CAF–PGE_2_ axis.

Factor	Potential Pathway	Evidence Type and Directness for Gastric Cancer
Chronic *H. pylori* infection	*H. pylori*-related inflammation → NF-κB/COX-2; CD38^+^ mast cells → adenosine metabolism → CD8^+^ T-cell suppression [42]	Indirect (gastric cancer mechanisms plus pan-cancer evidence)
EBV-negative/MSS subtype	EBV-negative: higher COX-2/PGE_2_ pathway activity and lower CD8^+^ infiltration; MSS: CAF enrichment and primary ICI resistance	Transcriptomic and clinical associations
Chemotherapy-induced CAF senescence	SASP release of PGE_2_, IL-6, and MMPs through the miR-335/PTEN axis	Primarily other-cancer and in vitro evidence
Hypoxia/inflammatory feedback	IL-1β/TGF-β/HIF-1α maintenance of PTGS2/mPGES-1; PGE_2_–COX-2 positive feedback; loss of 15-PGDH	Mechanistic evidence across cancer types

Note: Evidence strength is qualitative; the relative contribution and causality of each factor require direct validation in gastric cancer.

**Table 3:** Potential strategies for targeting the CAF–PGE_2_ pathway and supporting translational evidence.

Target/Strategy	Representative Intervention	Mechanism	Gastric Cancer Evidence	Cross-Cancer Evidence	Development Stage	Main Limitation
EP4 blockade	YY001; ONO-4578; E7046; ASP7657	Blocks EP4 and restores APC, CD8^+^ T-cell, and myeloid function	YY001 models; clinical signals from ONO-4578 plus ICI [29,38,39]	Supported in multiple models [82,83,84]	ONO-4578: phase II; E7046: phase I [85,86]	Does not establish CAF origin; predictive biomarkers are lacking
COX-2 inhibition	Celecoxib; NSAIDs; selective PTGS2 inhibition	Reduces PGE_2_ synthesis and relieves immune suppression	COX-2 is associated with PGE_2_/prostaglandin synthesis and angiogenesis [87]; limited CAF-specific functional evidence	Supported in multiple models [26,88,89]	Exploratory combinations; no CAF-based stratification	Source-nonspecific; long-term safety
mPGES-1 inhibition	mPGES-1 inhibitors; CAF-intrinsic mPGES-1 inhibition	Selectively blocks terminal PGE_2_ synthesis	Insufficient direct evidence in gastric cancer; CAF PTGES requires localization	Supported in multiple models [9,90,91]	Preclinical; no gastric cancer ICI combination	Cellular source must be resolved
Biomarkers and patient selection	PTGS2/PTGES/EP4; PGE_2_ metabolites; CAF signature plus spatial CD8^+^ exclusion	Combines source, pathway activity, and spatial state	Exploratory benefit signal in the CPS ≥ 1 subgroup of ONO-4578-08; CAF/PGE_2_ status not validated [39]	CAF signatures support candidate stratification [5,77,78]. Additional CAF subtype signals remain exploratory [79,80,81]. EP4-based evidence is also exploratory [92].	Exploratory; prospective validation required	Not a companion diagnostic

## Data Availability

No new data were created or analyzed in this study. Data sharing is not applicable to this article.

## Supplementary Materials

The following supporting information can be downloaded at: https://www.mdpi.com/article/10.3390/ijms27219544/s1.
