# Supplementary material for: Cancer-Associated Fibroblasts and Prostaglandin E2 Signaling in Gastric Cancer: Immunometabolic Mechanisms and Implications for Immunotherapy Resistance
Source: Int J Mol Sci. 2026 Oct 25;27(21):9544. doi: 10.3390/ijms27219544 (PMC13763051; doi:10.3390/ijms27219544)
Supplement: Supplementary file 1 [file ijms-27-09544-s001.zip › ijms-4597807-supplementary.pdf]

Supplementary Material

Supplementary Box S1. Falsification criteria for the CAF – PGE_2 model

A negative result is informative only when target engagement is confirmed, the intervention is sufficiently source-specific, statistical power is adequate, the relevant time window is covered, compensatory pathways have been considered, and the finding is reproducible. When a mechanism is untested or the methods are insufficient, it should remain a candidate.

Criterion	Potentially informative negative result	Interpretation
1. Source	With the required validity criteria met, tissue PGE_2 remains unchanged after CAF-specific PTGS2/PTGES intervention	Argues against CAFs as a major source; tumor and myeloid sources should be compared
2. Spatial association	With adequate sampling and spatial sensitivity, no reproducible association is observed between the candidate PGE_2-competent CAF program and $CD8^+$ exclusion	Argues against a specific PGE_2-related CAF immune niche; an untested association remains a candidate
3. Mediation	Source-specific blockade lowers tissue PGE_2, but immune or ICI phenotypes remain reproducibly unchanged and rescue fails	Strongly argues against a necessary mediator; partial improvement would support partial mediation
4. Competing mechanisms	In a sufficiently powered joint model, ECM/lactate/CXCL12 explain the effect and PGE_2 provides no incremental contribution	More consistent with an accompanying marker or the parallel-amplification end of the continuum
5. Clinical prediction	External validation shows no incremental predictive benefit and the confidence interval excludes a clinically meaningful improvement	Limited biomarker utility; does not exclude a biological effect

Supplementary Table S1. Evidence for PGE_2 production by gastric cancer CAF populations and therapeutic implications

CAF population	PGE_2-related molecular features	Direct evidence in gastric cancer	Evidence from other tumors/models	Therapeutic implication
iCAF	High PTGS2, IL-6, IL-8, and CXCL12	Single-cell/transcriptomic studies associate iCAF enrichment with immune suppression and ICI nonresponse; population-level PGE_2 quantification remains limited	Inflammatory/COX-2 programs are expressed by CAFs in pancreatic, breast, and other cancers; fibroblast PGE_2 sources are supported in additional models	Selective inactivation or reprogramming of iCAFs; combination with EP4 blockade
Senescent CAF	SASP components include COX-2/PGE_2, IL-6, and MMPs; miR-335/PTEN axis	Direct evidence for therapy-induced CAF senescence and its PGE_2 contribution in gastric cancer remains to be established	The COX-2 – PGE_2 – miR-335 – PTEN – SASP axis is supported in oral squamous cell carcinoma and in vitro models	Optimize chemotherapy timing; consider reversal of SASP with COX-2 inhibition
myCAF	ACTA2, TAGLN, COL1A1,	Associated with ICI nonresponse and $CD8^+$ T-cell exclusion;	$COX\text{-}2^+$ CAFs are associated with poor outcome and	Reduce ECM stiffness or reprogram

CAF population	PGE_2-related molecular features	Direct evidence in gastric cancer	Evidence from other tumors/models	Therapeutic implication
	POSTN; relatively low PTGS2	$POSTN^+$ CAFs are linked to $CD8^+$ T-cell exhaustion	invasion in nasopharyngeal carcinoma	selected CAF states; these findings do not establish PGE_2 mediation
apCAF	HLA-DR, CD74, C3	Potentially bidirectional roles in gastric cancer require validation	Complement secretion may regulate immunity; antigen presentation is possible	Preserve or induce antigen-presenting activity where appropriate
Perivascular/matrix-associated CAF (vCAF)	CD146, THBS1, THBS2, COL4A1	Recruits tumor-associated neutrophils and promotes $SPP1^+$ tumor-associated macrophage accumulation; direct links to PGE_2 are insufficient	Reviews support proangiogenic and immunosuppressive functions of vCAFs in other tumors	Target stromal – myeloid niches

Supplementary Table S2. Candidate multiparameter biomarker framework for the CAF – PGE_2 pathway

Candidate component	Definition	Detection method	Evidence level
CAF-related features	iCAF/senescent CAF expression programs indicating PGE_2 synthetic potential	Single-cell RNA-sequencing signatures; mPGES-1 immunohistochemistry	Partial (transcriptomic)
PGE_2 metabolic burden	Measured tissue PGE_2 level	LC – MS/MS quantification; COX-2/mPGES-1 score	Limited
Spatial relationship	Distance and distribution between CAFs and $CD8^+$ T cells	Multiplex immunofluorescence; CODEX	To be established

Note: This candidate framework integrates CAF-related features, PGE_2 pathway activity, and spatial immune states. No model, feature weights, thresholds, or predictive performance have been validated. It is not suitable for clinical decision-making and requires prospective validation in ICI-treated cohorts.

Supplementary Table S3. Representative study-level evidence matrix for the CAF－PGE_2－ICI model

Note: This is a representative rather than systematic or exhaustive list. Studies were selected to cover key evidence nodes, cellular sources, interventions, PGE_2 readouts, immune or ICI endpoints, and competing mechanisms. ‘Not measured’ indicates an untested variable and is not negative evidence.

Cancer type	Dataset/model	Cell source	Intervention	PGE_2 readout	Immune endpoint	ICI endpoint	Causal level supported	Main limitation
GC	Patient-derived gastric fibroblasts; in vitro	Gastric fibroblasts	Selective COX-2 inhibition	Culture PGE_2	Not primary	None	CAF source competence	No in situ source fraction or ICI endpoint
GC	Clinical cohort plus organoid/co-culture	PDPN+ CAF	Functional co-culture	Not measured	Immune activation	Neoadjuvant response association	CAF→ICI/function	Does not identify PGE_2 mediation
GC	CAF/tumor models	CAF	Lactate-pathway perturbation	Not measured	PD-L1/immune evasion	Anti-PD-1 phenotype	CAF→ICI through lactate	Competing non-PGE_2 mechanism
GC	Peritoneal-metastasis models	POSTN+ CAF	ICAM-1-related perturbation	Not measured	$CD8^+$ exhaustion	Not definitive	CAF→immune dysfunction	Competing non-PGE_2 mechanism
GC	Preclinical gastric cancer models	Receptor level; mixed source	EP4 antagonist YY001	Not source-resolved	APC/T-cell/M1 infiltration	Anti-PD-1 combination	PGE_2 receptor node→ICI	Cannot assign a CAF source
G/GEJ	ONO-4578 clinical studies	Receptor level; source unknown	EP4 antagonist plus nivolumab with or without chemotherapy	Not measured	Clinical only	Response/PFS	Clinical pathway tractability	No CAF/PGE2 biomarker; combination design
Neuroblastoma	CAF model	CAF	mPGES-1 inhibition	PGE_2	Tumor/immune context	None	CAF-derived PGE_2 function	Different cancer; no ICI endpoint
Lung	Fibroblast premetastatic-niche model	Lung fibroblasts	Fibroblast/PGE_2 manipulation	PGE_2	Immune microenvironment	None	Fibroblast-derived PGE_2 mechanism	Non-GC; not a CAF–ICI closed loop
Cross-cancer	Fibroblast-specific genetic model	Fibroblasts	IGF2 loss	Not measured	T-cell exclusion/function	ICI response	CAF→ICI causality	Competing non-PGE_2 route
Cross-	CAF-rich	CAF	NOX4	Not	$CD8^+$	ICI	CAF→ICI	Competing

Cancer type	Dataset/model	Cell source	Intervention	PGE_2 readout	Immune endpoint	ICI endpoint	Causal level supported	Main limitation
cancer	tumor models		inhibition	measured	exclusion	response	causality	non-PGE_2 route
Cross-cancer	Fibroblast perturbation models	Fibroblasts	AEBP1 blockade/loss	Not measured	T-cell dysfunction	ICI response	CAF→ICI causality	Competing non-PGE_2 route
Melanoma	Tumor-cell genetic model	Tumor cells	PTGES/mPGES-1 loss	PGE_2	$CD8^+$ T cells and $CD8\alpha^+$ DCs	Anti-PD-1 response	PGE_2→ICI; tumor source	Shows source substitutability, not a CAF source
Lung	KRAS tumor models	Tumor cells	Genetic/pharmacologic COX-2 blockade	PGE_2 pathway	Myeloid/$CD8^+$ state	ICI response	PGE_2→ICI; tumor source	Non-GC and non-CAF source
CRC	Myeloid reprogramming models	Myeloid/receptor level	EP4 antagonism	Not source-resolved	M2/MDSC and $CD8^+$ T cells	Anti-PD-1 response	EP4 immune mechanism	Does not establish the producing cell
Cross-cancer	CD8-specific genetic signaling model	$CD8^+$ T cells	Gαs–PKA activation	Not required	T-cell dysfunction	ICI failure	Downstream sufficiency	Bypasses PGE_2 source and receptor exposure
MSS CRC	TAM source model	TAM	Source/pathway perturbation	PGE_2	$CD8^+$ T-cell exhaustion	PD-L1 blockade	Myeloid PGE_2→ICI	Different cancer; alternative source
GC	Spatial immune-niche review	Mixed stromal/ immune compartments	None	Not source-resolved	Spatial immune organization	ICI resistance framework	Background synthesis	Review; no source-specific perturbation
GC	CAF/tumor chemoresistance models	CAF	NRP2-related perturbation	Not measured	Not primary	Chemoresistance	CAF-mediated therapy resistance	No PGE_2 or ICI endpoint
GC	CAF subtype models	$C3^+$ CAF	NF-κB pathway perturbation	Not measured	Immune/tumor context	Therapeutic resistance	CAF-state mechanism	PGE_2 mediation not tested
GC	ER-stress CAF models	CAF	NEAT1/SEMA3A pathway perturbation	Not measured	Immunosuppression	Not definitive	CAF–immune mechanism	No PGE_2 readout
GC	CAF/exosomal-miRNA models	CAF	Vitamin D/miR-378c intervention	Not measured	Tumor-microenvironment remodeling	Not measured	CAF reprogramming	No PGE_2 or ICI endpoint
GC	Human COX-2 genetic evidence	Host/tumor pathway	Genetic association	COX-2 pathway	Inflammation	Not measured	Susceptibility association	Does not resolve cell source

Cancer type	Dataset/model	Cell source	Intervention	PGE_2 readout	Immune endpoint	ICI endpoint	Causal level supported	Main limitation
Gastric preneoplasia	H. pylori mouse model	Mixed gastric tissue	PGE_2 pathway manipulation	PGE_2	Inflammation/infection persistence	None	Context-dependent PGE_2 function	Pre-neoplastic model; no ICI endpoint
Cross-cancer	CAF activation models	CAF	PKM2/NF-κB perturbation	Not measured	Inflammatory CAF state	None	CAF activation mechanism	No PGE_2 or ICI endpoint
Cross-cancer	Mechanistic review	T cells	None	PGE_2 pathway	T-cell differentiation	Not primary	Background pathway synthesis	Review-level evidence
GC	NETs/COX-2 models	Mixed tumor/immune compartments	COX-2-pathway perturbation	COX-2 pathway	Immune microenvironment	Not measured	Gastric inflammatory mechanism	CAF source not assessed
GC	CAF–NK co-culture/models	CAF	Iron/ferroptosis pathway perturbation	Not measured	NK-cell cytotoxicity	None	CAF–NK functional mechanism	Competing non-PGE_2 mechanism
Prostate cancer	Tumor-cell ABCC4 models	Tumor cells	ABCC4 perturbation	PGE_2 release	$CD8^+$ T-cell dysfunction	PD-1 blockade	Tumor-source PGE_2–ICI	Non-GC and non-CAF source
Cross-cancer	Monocytic MDSC models	Myeloid cells	Adenosine-pathway depletion	Not measured	Myeloid suppression	Immunotherapy response	Competing myeloid mechanism	Not a PGE_2-source study
HCC	Tumor/macrophage models	Tumor and macrophages	COX-2 pathway perturbation	COX-2 pathway	CTL exhaustion/ M2 polarization	Not definitive	COX-2–myeloid mechanism	Non-GC; PGE_2 not source-resolved
GC	TAM/tumor invasion models	Tumor-associated macrophages	COX-2/MMP9 perturbation	Not measured	Invasion and macrophage state	None	Gastric myeloid mechanism	No ICI endpoint
Cross-cancer	Th17/myeloid tumor models	Th17 and myeloid cells	COX-2/PGE_2 pathway perturbation	PGE_2 pathway	CTL reinvigoration	Anti-PD-1 response	Myeloid PGE_2–ICI mechanism	Non-GC and non-CAF source
GC	Tumor-cell/TME models	Tumor cells	MFSD2A manipulation	Not measured	T-cell activation	Anti-PD-1 response	Competing ICI-resistance mechanism	No CAF/PGE_2 mediation
CRC	Drug-treatment tumor models	Tumor/TAM compartments	Cox2/PGE_2 modulation	PGE_2 pathway	TAM infiltration	Anti-PD-L1 response	PGE_2-pathway intervention	Non-GC; source not resolved

Cancer type	Dataset/model	Cell source	Intervention	PGE_2 readout	Immune endpoint	ICI endpoint	Causal level supported	Main limitation
Cross-cancer	CAF-subtype models	CAF	CAF-subtype comparison	Not measured	NK-cell function	Not primary	CAF–NK mechanism	No PGE_2 or ICI endpoint
NSCLC	Narrative review	CAF	None	Not measured	Immune regulation	ICI context	Background CAF synthesis	Review-level, non-GC evidence
Breast cancer	CAF reprogramming models	CAF	Curcumin treatment	Not source-resolved	Inflammation/CAF state	Not primary	CAF reprogramming	Non-GC; ICI endpoint absent
Cross-cancer	Tumor-cell P2RY6 models	Tumor cells	P2RY6 perturbation	PGE_2 production	Immunosuppression	Not definitive	Tumor-source PGE_2 mechanism	Non-GC and non-CAF source
Cross-cancer	Medicinal-chemistry models	EP4 receptor level	EP4 antagonists	Not source-resolved	Immune activation	Cancer-immunotherapy development	Pharmacologic tractability	Does not identify PGE_2 source
Cross-cancer	Mechanistic commentary/review	Mixed compartments	COX-2 pathway	PGE_2 pathway	Anticancer immunity	ICI context	Background pathway synthesis	Review-level evidence
GC	CLEVER-1 blockade models	Myeloid/stromal compartment	CLEVER-1 blockade	Not measured	Immune evasion	Anti-PD-1 response	Competing ICI-resistance mechanism	No CAF/PGE_2 mediation
GC	Spatial immune-barrier analysis	$SPP1^+$/$C1QC^+$ macrophages	None	Not measured	$CD8^+$ T-cell exhaustion	ICI resistance	Stromal–myeloid association	No PGE_2 source measurement

Supplementary Table S4. Comparison of potential cellular sources of PGE_2 in the gastric cancer microenvironment

PGE_2 source	Strength of available evidence	Main limitation
Tumor cells	Strong evidence for a COX-2/PGE_2 pathway across multiple solid tumors	Limited spatial localization and stromal–immune interaction data
Myeloid cells	Strong association with inflammation and immune suppression in MDSCs and TAMs	High population heterogeneity and difficulty quantifying source contribution
CAFs	Stromal localization, potential for reprogramming, and spatial proximity to $CD8^+$ T cells	Insufficient direct quantification in gastric cancer; a prerequisite for source attribution

Note: Available evidence does not quantify the CAF fraction of total intratumoral PGE_2; this table supports CAFs as a candidate, not dominant, source.
